# Supplementary figures and images for: Integrative analysis of miRNA–mRNA network in idiopathic membranous nephropathy by bioinformatics analysis
Source: PeerJ. 2021 Sep 29;9:e12271. doi: 10.7717/peerj.12271 (PMC8487241; doi:10.7717/peerj.12271)

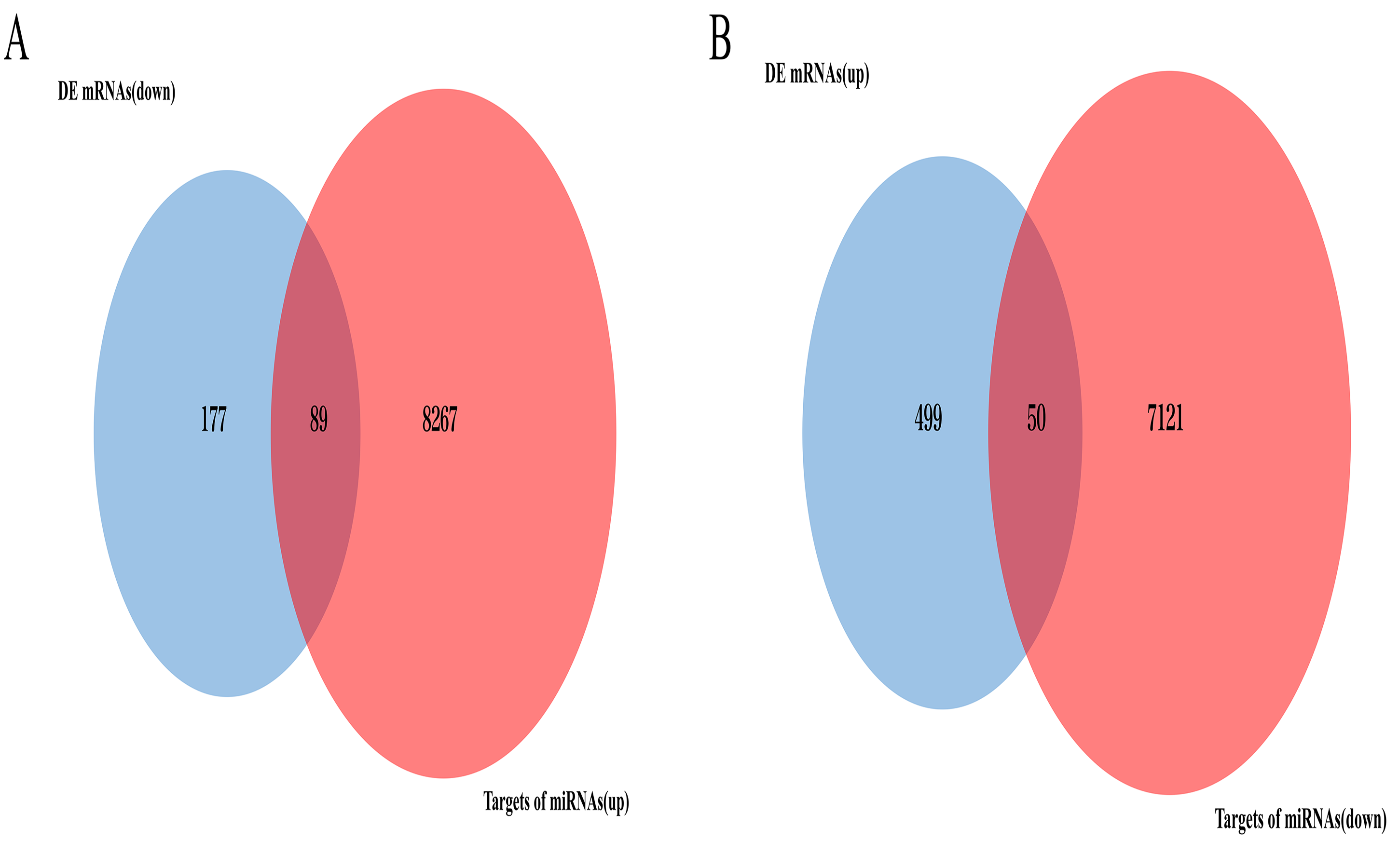

Supplement: Supplemental Information 1 — (A) VENN plot analysis for targets of upregulated DE miRNAs and downregulated DE mRNAs. (B) VENN plot analysis for targets of downregulated DE miRNAs and upregulated DE mRNAs [file peerj-09-12271-s001.png]

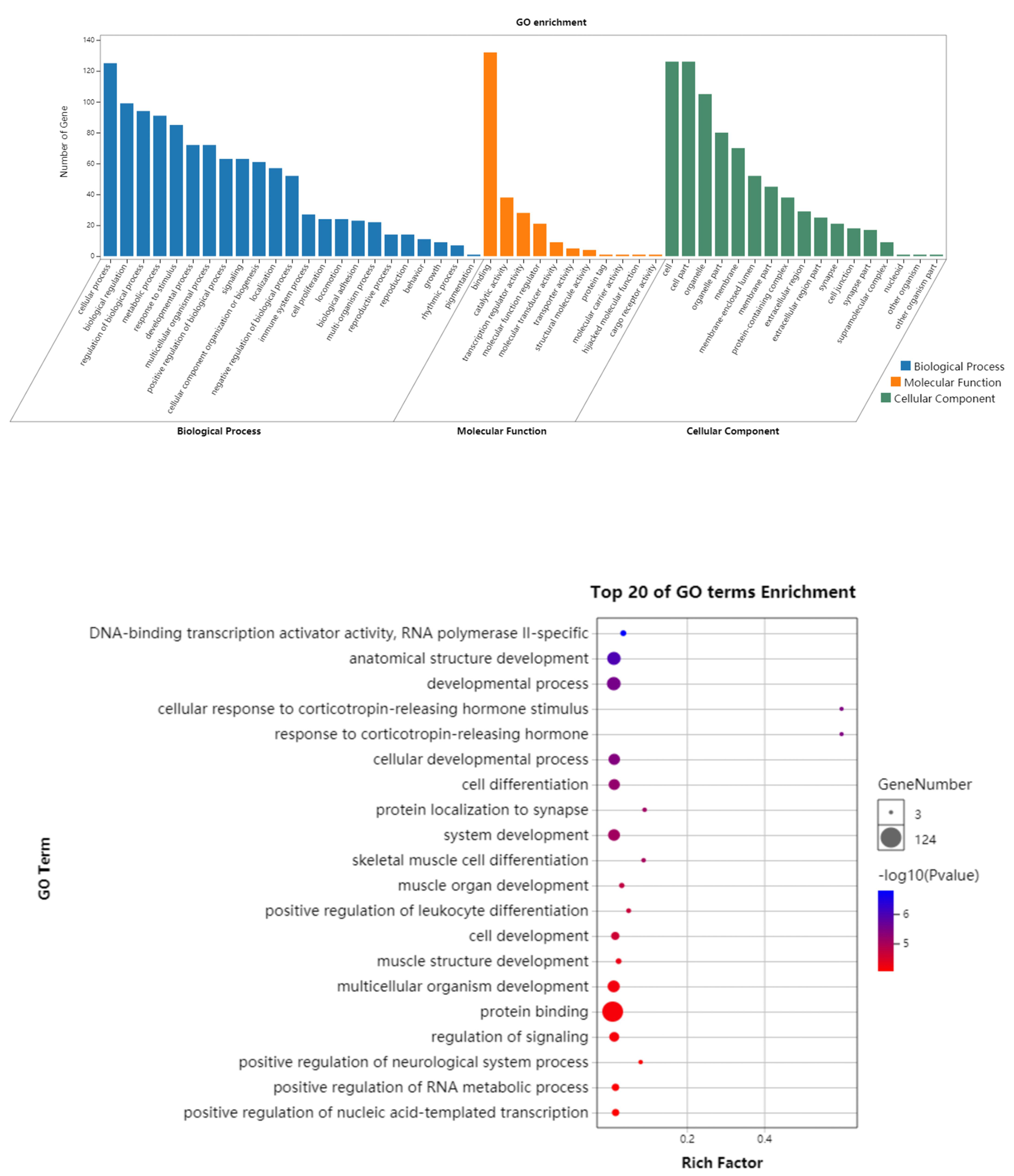

Supplement: Supplemental Information 2 — (A) Results of GO enrichment analysis of the key genes in the regulatory network. The blue yellow and green blocks represent the biological process cellular component and molecular function. The length of the blocks represents the number of genes enriched by a certain item. (B) Top 20 identified GO enrichment of key genes in the regulatory network. The node size represents the number of genes enriched in a certain pathway. The deeper the color, the smaller the p-value was. [file peerj-09-12271-s002.png]

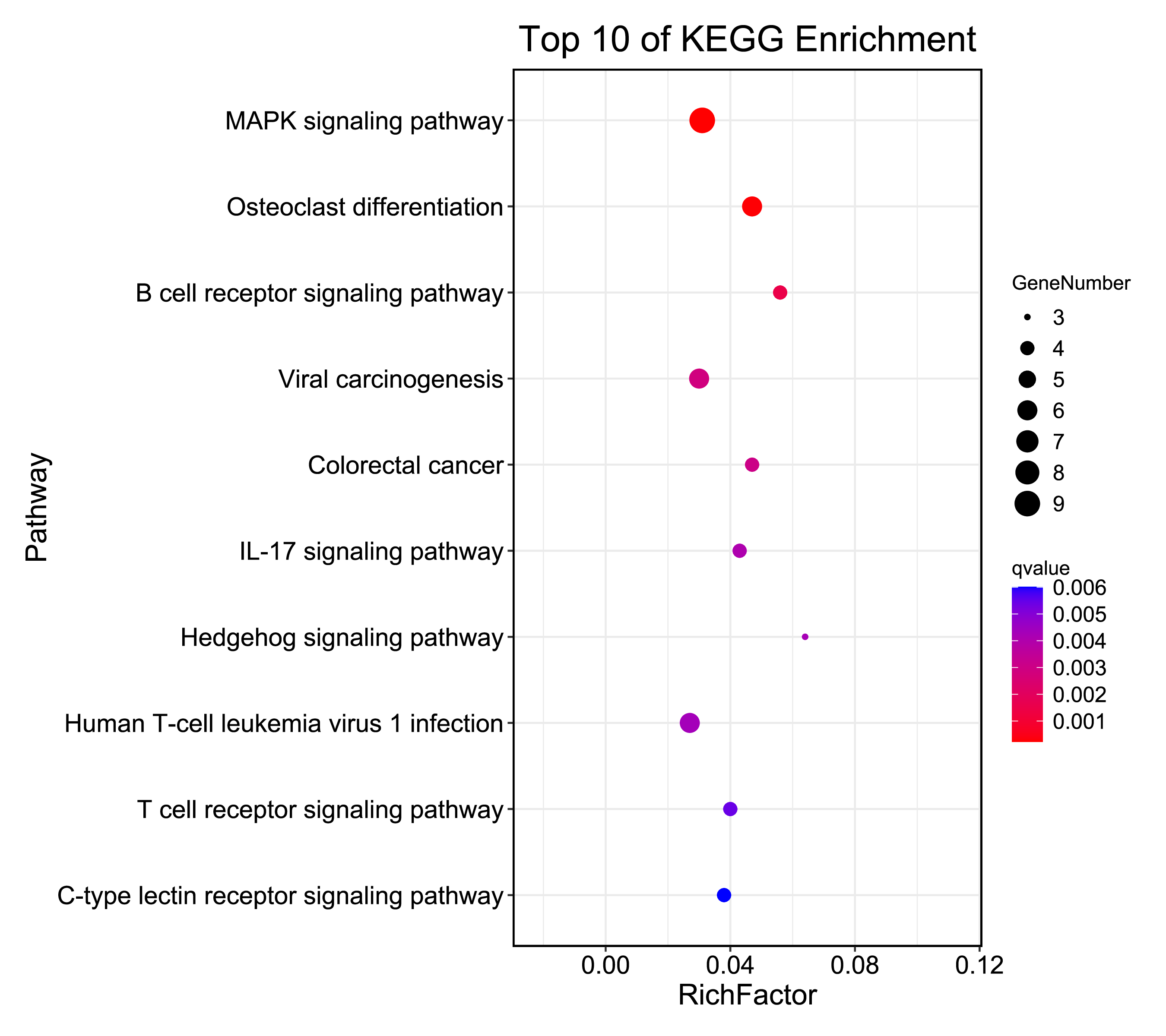

Supplement: Supplemental Information 3 — The node size represents the number of genes enriched in a certain pathway. The deeper the color, the smaller the p-value was. [file peerj-09-12271-s003.png]
